# Administration of adipose-derived mesenchymal stem cell conditioned medium improves ovarian function in polycystic ovary syndrome rats: involvement of epigenetic modifiers system

**DOI:** 10.1186/s13048-023-01317-9

**Published:** 2023-12-15

**Authors:** Golnaz Shafiei, Mona Saheli, Sepideh Ganjalikhan-hakemi, Tahereh Haghpanah, Seyed Noureddin Nematollahi-mahani

**Affiliations:** 1 Anatomical Sciences Department, Afzalipour School of Medicine, Kerman University of Medical Sciences, Kerman, Iran; 2 Physiology Research Center, Institute of Neuropharmacology, Kerman University of Medical Sciences, Kerman, Iran

**Keywords:** Adipose-derived mesenchymal stem cells, DNA methylation, Histone deacetylases, Estrogen receptor, Polycystic ovary syndrome

## Abstract

**Background:**

Polycystic ovary syndrome (PCOS) is a widespread heterogeneous disease that is in association with genetic, epigenetic, endocrine and environmental factors. Adipose-derived mesenchymal stem cell (ASC) and ASC-conditioned medium (ASC-CM) have shown promising abilities in tissue regeneration. In the present study, we aimed to investigate the effects of ASC and ASC-CM on epigenetic regulators, steroidal function and folliculogenesis in the letrozole-induced PCOS rats.

**Results:**

Based on the measurement of the oral glucose tolerance test and physical parameters including body weight, estrus cycle pattern as well as ovary dimensions, PCOS-induced rats in sham and control (CTRL) groups showed signs of reproductive dysfunctions such as lack of regular estrus cyclicity, metabolic disorders such as increased ovary dimension, body weight and blood glucose level alteration which were improved especially by ASC-CM administration.

**Supplementary Information:**

The online version contains supplementary material available at 10.1186/s13048-023-01317-9.

## Introduction

Polycystic ovary syndrome (PCOS) is one of the most important diseases related to the ovarian problems, affecting 6–15% of women of reproductive age [1, 2]. PCOS involves a series of symptoms, such as weight gain, menstrual irregularities, ovulation disorder, etc., that are caused by excess androgen production. Furthermore, disruption of the hypothalamus-pituitary–gonadal axis, folliculogenesis, insulin production and sensitivity, hormone profile, and estrogen synthesis may result from increases in androgens [3].

Steroid hormones affect some ovarian functions via autocrine or paracrine pathways via estrogen receptors (ESRs) [4]. ESRs (ESRα and ESRβ) are expressed in granulosa cells (GCs), and theca cells (TCs) in developing follicles [5]. ESRβ activation stimulates follicular growth, and reduces atresia [5]. In contrast, ESRα activation inhibits the ovulation probably via affecting the hypothalamic–pituitary axis and uterine growth. The expression levels of these receptors in the cumulus cells (CCs) of patients with PCOS are significantly lower than those of healthy controls [6]. Previous studies showed that epigenetic changes play a definite role in the steroidogenic activity in GCs and TCs of follicles [7].

Epigenome changes, such as DNA methylation, histone modifications, and non-coding RNAs, now play an important role in defining the etiology of PCOS [8]. DNA methylation is accomplished by DNA methyltransferase enzymes (DNMTs), which are divided into three classes in mammals: DNMT1, DNMT3A, and DNMT3B. The acetylation of histones is one of the main types of histone modifications that occur by the histone acetyltransferases (HATs), and histone deacetylases (HDACs) enzymes [9]. Epigenetic changes may lead to abnormal gene expression, thereby predisposing individuals to develop PCOS [10]. Pathways related to adipogenesis, inflammation, glucose control, and immunological function showed aberrant patterns in the adipose tissue and skeletal muscles of PCOS patients when DNA methylation and transcriptional patterns were analyzed [11, 12]. Furthermore, such changes were reported in DNA methylation and transcriptional patterns of the ovary [13], GCs [14], CCs [7], and TCs [15] in PCOS patients. However, little is known about the effects of ovarian epigenetics modifications on the pathophysiology of PCOS and there is still no effective treatment for PCOS. Currently, using stem cells, especially mesenchymal stem cells (MSCs), was suggested as a novel promising therapy for the restoration of ovarian function, and the regulation of steroidogenesis in terms of their paracrine-induced anti-inflammatory, anti-fibrotic, and angiogenic factors secreted by stem cells [16]. Reports show that transplantation of MSCs derived from the placenta [17], menstrual blood [18] and bone marrow [19] can improve ovarian structure, and function in a murine model of premature ovarian failure (POF) by inhibiting apoptosis and repairing DNA damage, resulting in germ cell protection [20].

Adipose-derived mesenchymal stem cell (ASC) are considered an ideal candidate in medicine in terms of their easy isolation, self-renewal ability, as well as their ability to differentiate and proliferate properly [21]. ASC secretes a range of growth factors, immunological regulators, and cytokines. Instead of developing into certain cells, they may also lessen free radicals by secreting antioxidant substances, which enhances ovarian function via their paracrine actions [21]. Besides, the potential risk of immune reactions and cancer development, has raised several safety concerns related to MSC’s transplantation [22]. Thus, poor engraftment and insufficient viability of transplanted cells restrict their therapeutic efficacy [23]. Conditioned medium (CM) rich in various stem cell-derived secreted factors, such as growth factors, cytokines, extracellular matrix proteins, lipids, messenger RNAs (mRNAs), regulatory miRNAs, secretomes, microvesicles, and exosomes were proposed as an alternative to MSCs, where applicable [24–26]. The positive effects of these factors have been reported in the treatment of male and female infertility [27–29]. Transplantation of MSCs, and MSC-CM into POF model ovaries has improved the microenvironment, ovarian reserve, and performance, as well as fertility rate by reducing apoptosis in GCs and ovarian interstitial fibrosis [30]. Furthermore, the in vitro maturation (IVM) rate of oocytes cultured in media containing ASC-conditioned medium (ASC-CM) was higher than that of ASC alone [31].

Since no data are available to compare the effects of ASC and ASC-CM transplantation in the PCOS model, we aimed to investigate the effects of ASC and ASC-CM transplantations on epigenetic alterations, folliculogenesis, and estrogenic activity in the ovaries of PCOS model *Wistar* rats.

## Methods and materials

### Animals and maintenance method

In this experimental study, adults (180–220 g) female *Wistar* rats were purchased from the animal house of Afzalipour School of Medicine, Kerman, Iran. The experiments were performed with the permission of the ethics Committee of Kerman University of Medical Sciences (approval number: IR.KMU.AH.REC.1400.195) and the animals were maintained under standard laboratory conditions (12/12 h cycle of light and dark; at 22- 25 ^◦^C). After 10 days of acclimatization, a daily vaginal smear was prepared and only those animals that had at least two consecutive normal estrus cycles were included in the experiment [19].

### Experimental design

Sixty female *Wistar* rats were randomly divided into two groups: the carboxymethyl cellulose (CMC) group which received a 1% aqueous solution of CMC orally once daily (vehicle group, *n* = 10) and the PCOS groups, which received 1 mg/kg letrozole (Tehran, Iran) in 1% CMC orally once daily (PCOS group, *n* = 50) for 21 days [32]. After confirming the PCOS model, the animals in the PCOS group were divided into four subgroups: 1- Adipose mesenchymal stem cell receiving group (ASC, *n* = 10) that 2 × 10^6^ passage 4 ASC suspended in 20 µl medium were transplanted into the ovaries of PCOS rat model [19, 33], 2- ASC-conditioned medium group (ASC-CM, *n* = 10) which received 20 µl ASC-CM into the ovaries of PCOS rat model, 3- control group (CTRL, *n* = 10) and 4- sham group (Sham, *n* = 10) which received 20 µl culture medium and normal saline into the ovaries of PCOS rat model, respectively.

Vaginal smears were obtained daily from the 10^th^ day to the last day of the experiment for each group and the smears were evaluated under a light microscope (Olympus IX51, Japan) after staining with hematoxylin [34]. Four weeks after transplantation [19], the animals were deeply anaesthetized with 40 mg/kg ketamine and 4 mg/kg xylazine, the blood was taken from the left ventricle and the serum was separated from the blood sample by centrifugation at 3,000 rpm for 15 min, and kept refrigerated for serum estradiol measurement then the ovaries were immediately removed for the rest of the experiments.

### Induction of PCOS model

To induce the PCOS model, female rats received 1 mg/kg letrozole dissolved in 1% CMC orally once a daily for 21 days (PCOS groups, *N* = 50). The CMC group received only 1% CMC (vehicle group, *N* = 10) [32]. The animals underwent daily vaginal smear examination to confirm PCOS. To check the prolongation of metestrus and diestrus phases was considered as the main sign of PCOS, then it was reinforced by positive intraperitoneal glucose tolerance test (IPGTT) and an increase in the number of ovarian cysts and decrease in the number of corpus luteum.

### Glucose tolerance test

On the last day of the PCOS induction and treatment, the animals were fasted for 12–14 h (8 pm to 8 am). The next morning, their blood was taken from tail veins. In order to evaluate glucose tolerance, rats received an i.p dose of 2 g/kg glucose and their blood glucose was measured at different time points (30, 60 and 120 min) using a standard glucometer (CareSens, i-SENS, Inc, South Korea). The area under the glucose tolerance curve (AUC) was calculated during the 120-min sampling period [35].

### Body weight and tissue sampling

The body weight of animals was recorded every week from the first day to the end of the treatment period using a digital scale (Sartorius, Japan). After the animals were sacrificed, the ovaries were collected, washed with normal saline, and the excess fat covering the ovaries was removed. Right ovaries were stored at − 80 °C for gene expression assays, while the left ovaries were weighed, and their length and width were measured using a digital caliper (Sartorius, Japan), and then fixed in 10% formalin in PBS for histomorphological and immunohistochemical evaluation. Gonadosomatic index (GSI) was calculated as (ovary weight/body weight) × 100. Weight gain was also calculated as ([final weight – initial weight] /initial weight × 100) [36].

### Serum estradiol assay

Four weeks after transplantation, a blood sample was collected from the heart of the anesthetized animals in the diestrus phase at 8–9 AM. [37]. The samples were centrifuged at 3000 rpm for 15 min. The serums were extracted and kept at -20 ºC until hormone analysis. 17-β estradiol was measured using a specific kit (Monobind, Inc., U.S.)and ELISA reader instrument. The intra-assay coefficient of variation (CV) of three replicates of each sample in a single assay was 8.1% and the inter-assay CV with analysis of the same samples (*n* = 6) in two separate runs was 9.3%.

### Ovarian follicle counts

The ovaries were dehydrated, paraffin-embedded, and serially sectioned at 5-μm thickness. From every 10 serial sections, one section was selected and stained with hematoxylin and eosin (eight sections from each rat). Folliculogenesis was investigated by determination of different types of follicles based on the following criteria: 1- primordial follicles: an oocyte surrounded by a layer of squamous follicular cells, 2- primary follicles: an oocyte surrounded by a layer of cuboidal GCs, 3- secondary follicles: an oocyte surrounded by several layers of cube-shaped GCs without cavities, 4- pre-antral follicle: an oocyte surrounded by several layers of cube-shaped GCs with several small cavities between the cells, 5- antral follicle: an oocyte surrounded CCs in a space filled with liquid and several layers of GCs, 6- pre-ovulatory follicle: an oocyte surrounded by CCs and a large cavity around the cumulus-oophores-complex (COC), 7- Ovarian cysts with large cavities surrounded by GCs and absence of a COC, and finally 8- corpus luteum, multilayered GCs and TCs in the periphery of the ovary [19, 38].

### Culture and identification of ASC and preparation of ASC-CM

ASC was obtained from a frozen cell batch in our laboratory at Afzalipour faculty of medical sciences, Kerman, Iran. Flow cytometry was used to characterize the mesenchymal origin of ASC by analyzing the expression of cell surface CD markers, namely CD34^−^, CD45^−^, CD90^+^, and CD105^+^. In addition, the ASC were differentiated into adipogenic (50 mg/ml indomethacin, 100 nM dexamethasone and 50 mg/ml ascorbic acid) and osteogenic (10 mM b-glycerophosphate, 10 nM dexamethasone and 50 mg/ml ascorbic acid) linage for 21 days. Half of the culture medium was refreshed every 3 days. Adipocyte-like cells and osteoblast-like cells were determined using Oil Red O and Alizarin Red S staining, respectively [39].

The harvested cells were cultured and expanded in Dulbecco modified Eagle medium (DMEM; Gibco, BRL) supplemented with 10% fetal bovine serum (FBS), 100 U/mL penicillin, and 100 mg/mL of streptomycin and incubated at 37˚C with 95% humidity and 5% CO2. Two days later, the culture medium was replaced with fresh medium to remove debris [39].

To obtain the ASC‐CM, 1 × 10^4^ cells/cm^2^ passages four to six were seeded in T75 tissue culture flask and after reaching to 80% to 90% confluence, the cells were washed three times with PBS. Then, they were incubated in a serum‐free DMEM culture medium for 48 h and the CM was collected, concentrated 10 times (10 ×) by centrifugation at 7500 rpm for 25 min at 4 °C using 3‐kDa molecular weight (MW) cutoff filter units (Millipore; Burlington, MA), and then stored at − 70 °C until use [40].

### Transplantation and tracking of ASC

ASC (2 × 10^6^ at passage 4 to 6) were washed twice with PBS, pre-labeled with a red fluorescent dye; PKH26 (Sigma, US), suspended in 20 μl culture medium and then directly injected into the bilateral ovaries of ASC group (*n* = 4) [19]. Four weeks after transplantation, paraffin-embedded sections were prepared and the labeled cells were visualized by fluorescence microscopy (Olympus IX71, Japan) [41].

### Quantitative reverse transcriptase–polymerase chain reaction (qRT-PCR)

The mRNA expression levels of the main enzymes involved in the epigenetic modification (DNMT1, DNMT3A, DNMT3B, HDAC1 and HDAC2) and estrogen receptors (ESRα and ESRβ) were evaluated using quantitative RT-PCR. Briefly, total RNA was extracted from frozen right ovary using TRIzol (CINNAGEN, Iran) according to the manufacturer’s protocol [42]. The quantity and quality of the extracted RNA were determined using NanoDrop 2000 spectrophotometer (Thermo fisher scientific, Wilmington, DE, United States) and electrophoresis, respectively. Complementary DNA (cDNA) was synthesized from total RNA (1 μg) by a thermocycler (BIOMETRA, Germany) using a reverse transcription kit according to the manufacturer's protocol. To evaluate the expression levels of the different genes, 1 μl of the synthesized cDNA and 6 μl SYBR green master mix (Genaxxon bioscience, Ulm, Germany) were used in a total reaction volume of 10 μl using a Light Cycler Real-Time PCR System (MIC, Queensland, Australia). The sequences of the specific primers used are listed in Table 1. The relative expression of each gene was determined and normalized to the expression of housekeeping gene beta actin (β-actin), and calculated using the 2^−ΔΔCT^ method.

**Table 1:** List of specific primers used in real-time polymerase chain reaction assay

Gene name	Accession no	Designed oligonucleotide (5’ → 3’)	Product size (bp)
DNMT1	NM_053354.3	F: CAAGATGCCAGCACGAACAG R:AGCCATCTCTTTCCAAGTCTTTG	110
DNMT3A	XM_039112666.1	F:CGGATAATACCTTCTCTGAAGCCC R: CCTGTTCCTCTCCTTCCTTTCG	162
DNMT3B	NM_001396349.1	F:AGAAGAGGGTGCTAGTGGGTATG R: TTCATCTCCATCATCCGCTTCAC	250
HDAC1	NM_001025409.1	F: CCAGAAGCCAAAGGGGTCAAA R: TGTGCGCTGGTCCCTATCTAG	182
HDAC2	NM_053447.1	F: GAGGCGGCAAGAAGAAAGTGT R: GTCATCCGGATCCTATGGGG	100
ESRα	NM_012689.1	F: GAGCACATTCCTTCCTTCCGT R: AGGCTTTGGTGTGAAGGGTC	191
ESRβ	NM_012754.3	F: CGTTCTGGACAGGGATGAGG R: GCAGAAGCCAAGGGGTACAT	168
β-actin	NM_031144.3	F: CCCGCGAGTACAACCTTCTT R: CGCAGCGATATCGTCATCCA	83

### 5-methylcytosine (5mC) and 5-hydroxymethylcytosine (5hmC) assessment by immunohistochemistry

The right ovary was processed, embedded in paraffin, and cut into 5 μm sections to assess the protein expression of 5*-*methylcytosine (5mC) and 5-hydroxymethylcytosine (5hmC) as epigenetic markers. Briefly, the sections were deparaffinized in xylene and rehydrated in decreasing concentrations of ethanol (100, 95, 80, and 70). Then, antigen retrieval was performed in 0.01 M citrate buffer (pH 6.0) and high microwave irradiation for 25 min, followed by endogenous peroxidase blocking using TBS/H_2_O_2_ at room temperature for 30 min. The slides were washed three times in PBS-tween 20 (0.05%) for 5 min, and permeabilized in 0.5% triton for 10 min. Thereafter, they were incubated with a rat anti-5mC monoclonal antibody (RM231; cat.no. ab214727, Abcam, US, 1:40) and a rat anti-5hmC monoclonal antibody (RM236; cat.no. ab214728, Abcam, US, 1:40) overnight at 4 °C. After three washes in PBS for 20 min, the sections were incubated with biotinylated goat anti-rabbit IgG secondary antibody (cat.no. ab64261, Abcam, US, 1:40) for 1 h at room temperature. Finally, the slides were incubated with 0.5 mg/ml diaminobenzidine tetrahydrochloride 2-hydrate (DAB, Boster bio-engineering, USA) for 5 min. After washing in PBS for 10 min, they were mounted and protein expression of 5mC and 5hmC was visualized using a light microscope (Nikon, Japan) × 100 magnification. The intensity of images were analyzed using ImageJ Fiji software (version 1.52; WS Rasband, National Institute of Health, Bethesda, Rockville, MD, USA) based on a validated protocol [43].

### Western blot analysis of ESRα and ESRβ protein

Ovarian tissue of each sample (40 mg) was homogenized by lysis buffer (50 mM Tris, pH 7.5, 150 mM sodium chloride, 1% NP-40, 0.5% sodium deoxycholate, 0.1% SDS, 0.1 mM EDTA and 0.1 mM EGTA) supplemented with complete protease inhibitor cocktail (Roche, Mannheim, Germany). The samples were then centrifuged at 12,000 × rpm for 15 min at 4 °C and the supernatant was collected. We used the Bradford method to determine the protein concentration of the supernatant. Equal amounts of protein (10 µg) were incubated with a 2 × gel loading buffer and separated at 10% SDS-PAGE for two hours, before transferring to PVDF membranes (Roche, West Sussex, UK). The membranes were blocked with 5% skimmed milk in TBS-T. Afterward, blots were incubated overnight at 4 °C with primary antibodies including anti-ESRα, anti-ESRβ and anti-β-actin (Santa Cruz Biotechnology, USA, 1:300). The membranes were then incubated with HRP-conjugated anti-mouse and anti-rabbit secondary antibodies (Santa Cruz Biotechnology, 1:1000) for 1 h at room temperature, washed three times with TBS-T and visualized using the enhanced chemiluminescence (Pierce, USA) and exposed to X-ray films (Thermo Scientific). The gray value of each band was analyzed by gel image analysis software (ImageJ), then the ratio of the gray value of the target protein band to that of the internal reference protein band (β-actin) was calculated [44, 45]. 0.5—0.7 mg/ml was used for assays.

### Statistical analysis

Statistical analyses were performed using SPSS (version 16.0) and GraphPad Prism (ver 8.0.1, Graph-Pad Software Inc., San Diego, CA, USA) software. First, the normality of the variables was checked using the One-sample Kolmogorov–Smirnov test or the Shapiro–Wilk test and/or D’Agostino & Pearson test. If the study parameters had a normal distribution, an unpaired two-tailed Student’s t test was used (comparison between two experimental groups; PCOS and CMC groups) and the one-way ANOVA test (comparison between three or more experimental groups) followed by post hoc Tukey’s or, otherwise the nonparametric Kruskal–Wallis test or Mann–Whitney U test were run. Repeated-measures ANOVA was used in timewise comparisons. Data were expressed as mean ± SEM and *P* values ≤ 0.05 was considered statistically significant.

## Results

### PCOS rat model confirmation

To confirm the PCOS model, after the end of the 21-day treatment period, estrus cyclicity, ovarian histopathology, blood glucose levels at different times (0, 30, 60 and 120 min) and also AUC (glucose clearance rate as reflected by the area under IPGTT curves) were evaluated.

**Fig. 1:**
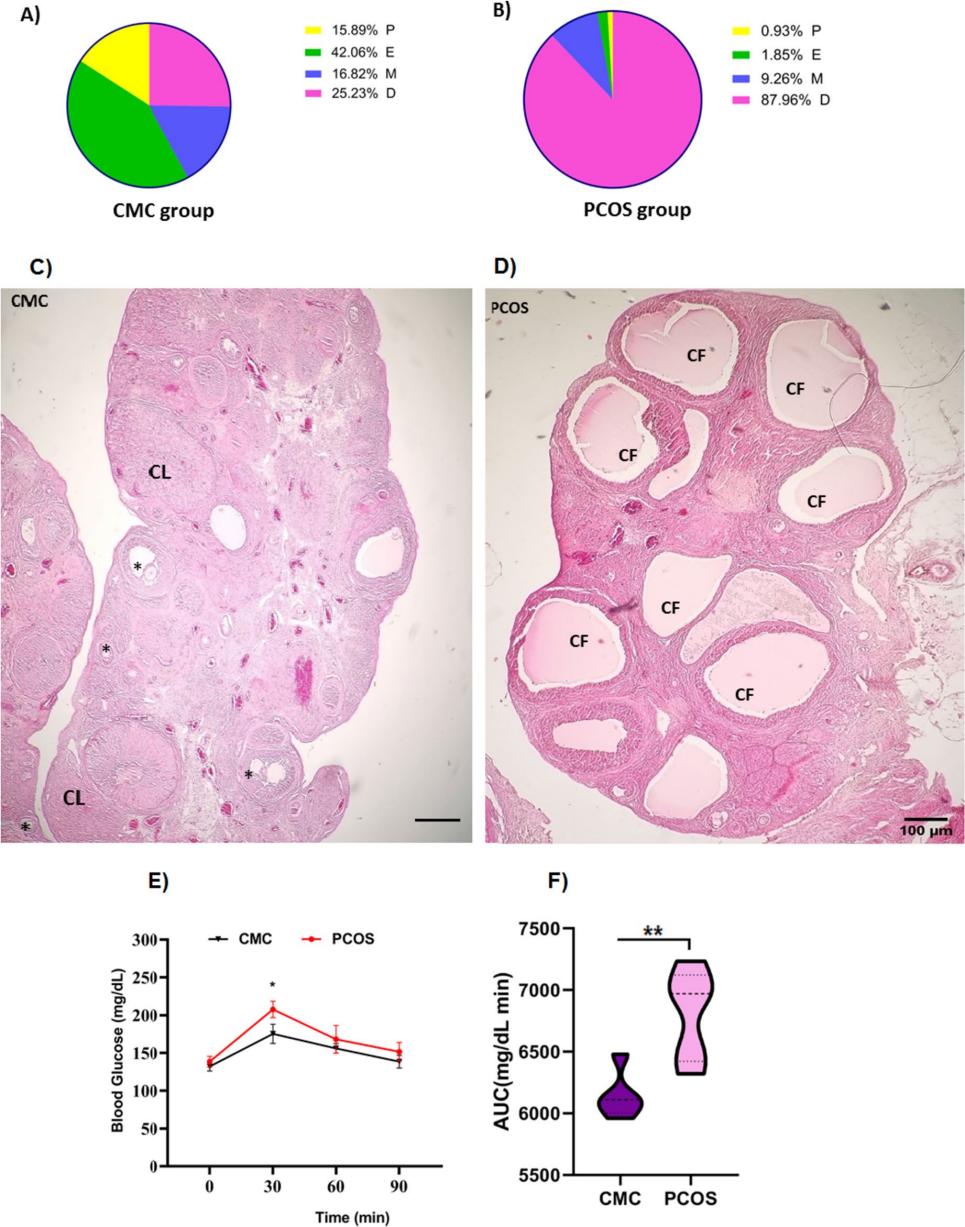
PCOS rat model characteristics: **A**, **B**; The pie charts refer to the proportions of the different stages of the estrus cycle: proestrus (P), estrus (E), metestrus/diestrus (M/D), **A** demonstrates a regular estrus cycle in the CMC group, and **B**) an irregular estrus cycle, confirmed by an increase in the duration of metestrus and distrus phases in the PCOS group. Histopathological tests: **C** presence of multiple corpus luteum (CL) and follicles at different developmental stages in the CMC group (stars), **D** presence of cystic follicles (CF) in the PCOS group. Intraperitoneal glucose tolerance test (IPGTT): **E** and glucose clearance rate and (**F**), the area under IPGTT curves (AUC) between the PCOS and CMC groups after 21 days of oral administration of 1 mg/kg letrozole or 1% CMC. In IPGTT curves **(E)**: * shows *P* ≤ 0.05, PCOS vs CMC group. Data are expressed as mean ± SEM*.* Repeated-measure ANOVA followed by Tukey’s post hoc test was used to analyze IPGTT data. unpaired two-tailed Student’s t test was used for AUC data analysis. Abbreviations: PCOS, polycystic ovary syndrome; CMC, Carboxymethylcellulose

Analysis of vaginal smears showed that the CMC group had a regular estrus cycle for 4 to 5 days, so that 25.23% of animals in the CMC group were in the diestrus phase. meanwhile, a failure of estrus cyclicity, as a critical characteristic of the PCOS rat model, was observed in PCOS rats, so that they spent 87.96% of their estrus cycle in the diestrus phase (Fig. 1.A, B).

Also, using histopathology evaluations, the corpus luteum and different developmental stages of follicles, which are indicative of folliculogenesis, were observed normally in the ovaries of the CMC group (Fig. 1.C), whereas multiple cystic follicles along with no signs of corpus luteum formation or ovulation were found in the ovaries of PCOS rats (Fig. 1.D).

Moreover, glucose concentration in the IPGTT was significantly different with time [F (2.257, 18.05) = 55.92, *P* < 0.0001] and group [F (1, 8) = 16.70, *P* = 0.0035]. Thirty minutes after glucose loading, the PCOS group had a higher blood glucose level in comparison with the CMC group (*P* = 0.0113) (Fig. 1.E). In addition, AUC was significantly higher in the PCOS group compared to the CMC group (*P* = 0.0080) (Fig. 1.F).

### ASC characteristics

**Fig. 2:**
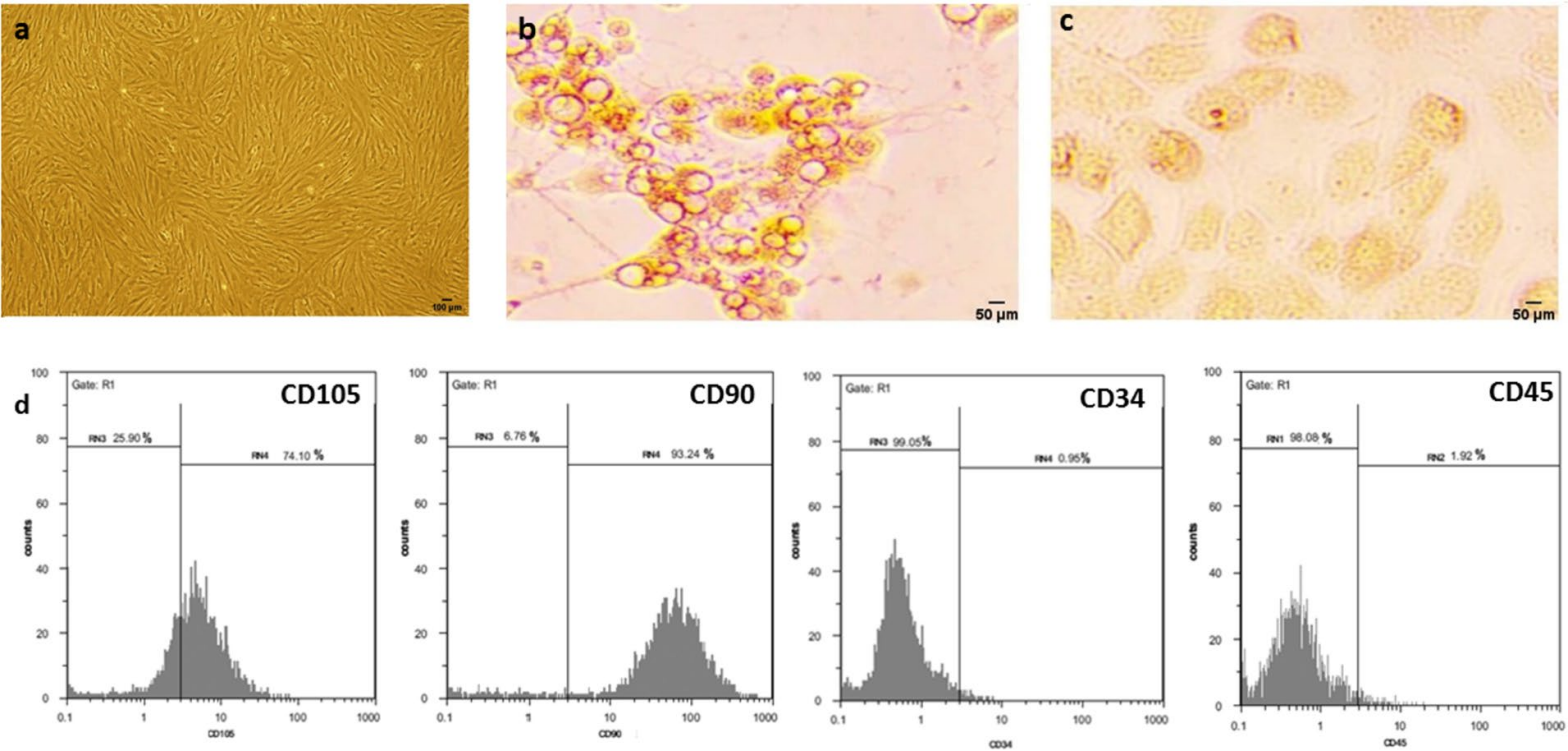
Characterization and differentiation of ASC. **a** Morphology of ASC at passage three. **b**, **c** ASC were able to differentiate into adipocytes (oil red O staining), and osteoblasts (alizarin red staining). **d** Flow cytometry analysis of phenotype characterization of the 3.^th^ passage ASC. which were positive for CD105, and CD90, and negative for CD34 and CD45

ASC propagated rapidly in vitro and was morphologically similar to fibroblast-like cells (Fig. 2.a). ASC were multipotent cells, as indicated by their ability to differentiate into adipocytes (round lipid vacuoles visualized with oil red O stain) and osteoblasts (extra cellular matrix mineralization stained red with alizarin red) (Fig. 2.b, c). ASC was positive for CD105 (74.10%), and CD90 (93.24%), and was negative for hematopoietic markers, namely, CD34 and CD45 (Fig. 2.d).

### Identification of transplanted ASC in the ovaries

**Fig. 3:**
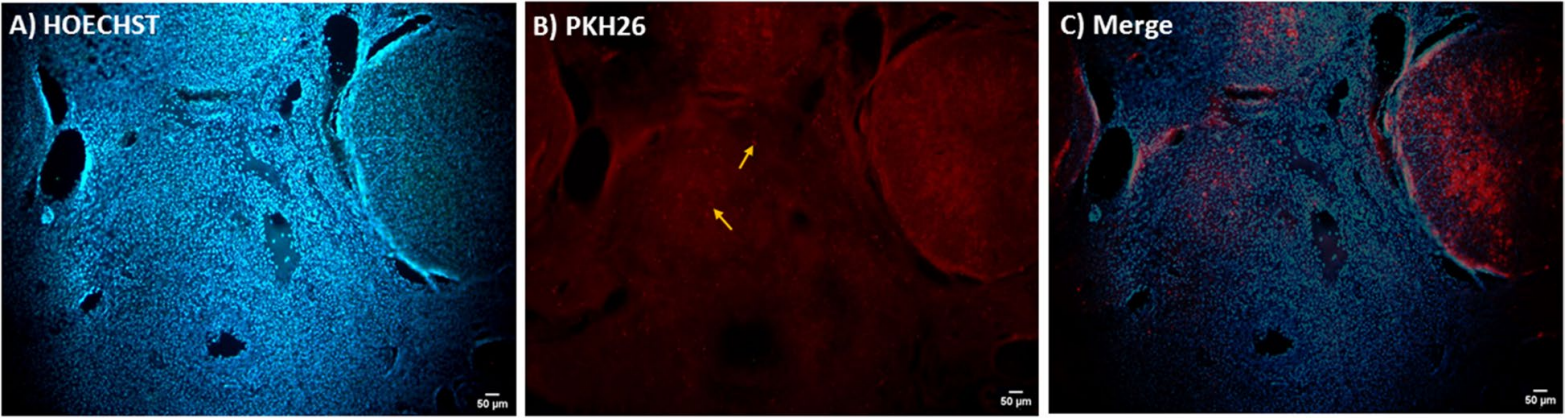
Presence of the ASC in the transplanted ovaries. **A** ovary section colored with Hoechst dye for nucleus visualization (blue spots). **B** PKH26-labeled ASC show red fluorescence in an ovary section (yellow arrows) **C**) Merged. Magnification, × 100

Fluorescent imaging showed that the PKH26-labeled ASC, were visible as red spots in the sections, four weeks after transplantation (Fig. 3).

### The effects of ASC and ASC-CM on body weight and ovarian morphometric parameters in PCOS rat model

**Fig. 4:**
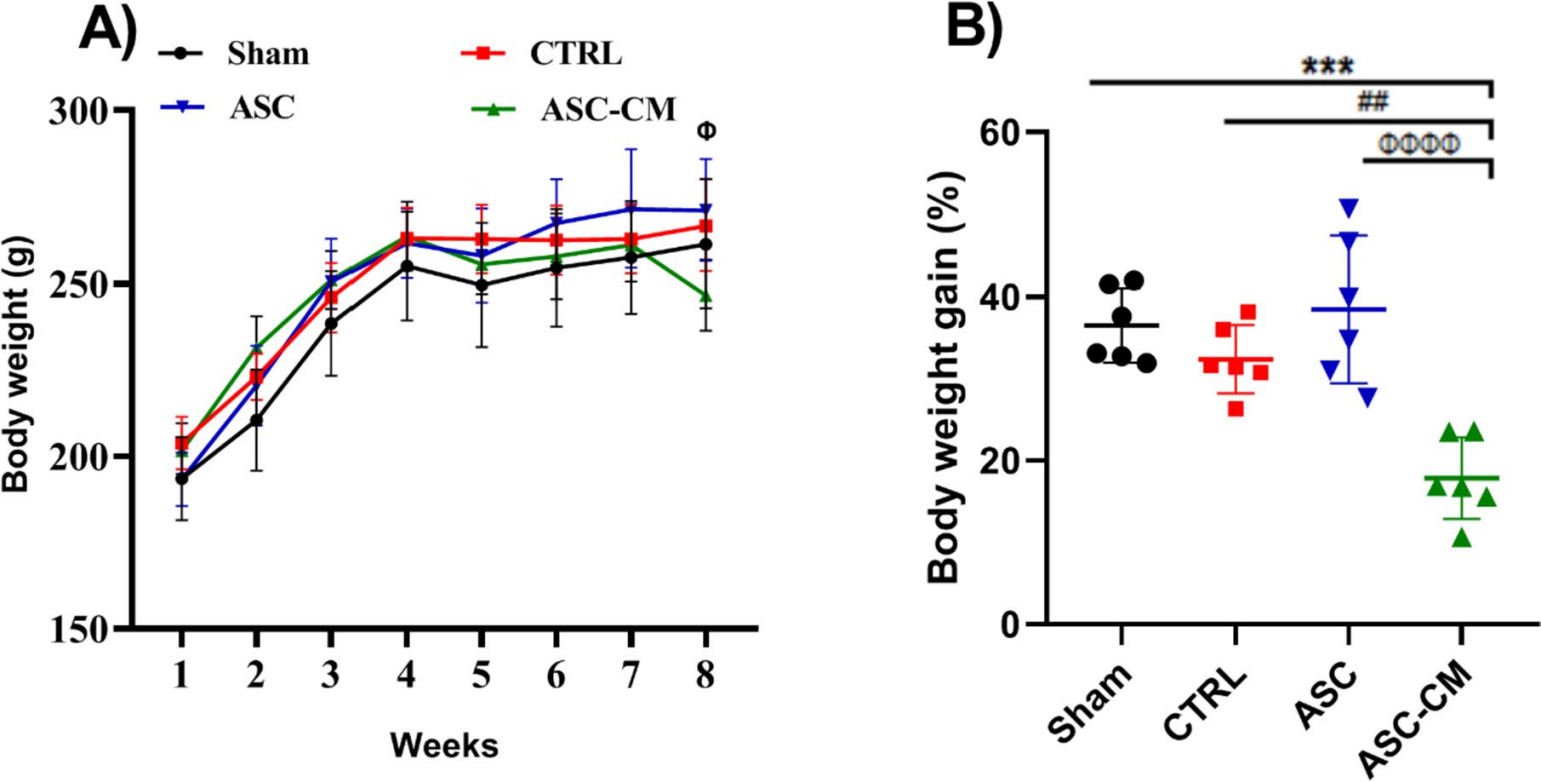
The effects of ASC and ASC-CM transplantation on the body weight (**A**), and weight gain (**B**). **A** ^Φ^: *P* ≤ 0.05, ASC vs ASC-CM. **B** *shows significant difference vs the sham group (*** *P* ≤ 0.001), ^#^ shows significant differences vs the CTRL group (^##^*P* ≤ 0.01). ^Φ^ shows significant differences vs the ASC group (^ΦΦΦΦ^*P* ≤ 0.000). Data are expressed as mean ± SEM. The body mass data were analyzed by a repeated measure two-way ANOVA (time, group) and Tukey’s post hoc test and the body weight gain data were analyzed by one-way ANOVA test. Abbreviations: CTRL, Control group; ASC, Adipose stem cell group; ASC-CM, Adipose stem cell-conditioned media group

Animals' body weight was measured weekly for 8 weeks. The two-way repeated measure ANOVA analysis revealed a statistically significant difference between the effects of group and time on body weight [F (21, 140) = 3.006, *P*<0.0001]. Our results showed that the ASC-CM group had markedly lower body weight than the ASC group 8 weeks after treatment (*P*= 0.0337; Fig. 4). Weight gain in the ASC-CM group was significantly decreased compared to the ASC (*P*= 0.0001), Sham (*P*= 0.0002), and CTRL (*P*=0.0024) groups (Fig. 4B).

Also, significant differences were observed in the ovary length and width among all groups, with the ovary length in the ASC-CM and ASC groups lower than the untreated groups (sham and CTRL groups) (*P*=0.0156, *P*=0.0106). The ovary width in the ASC-CM and ASC groups also significantly (*P*=0.0019, *P*=0.0090) was lower than the Sham group (Table 2). However, the ovary weight was higher in the ASC-CM group and significantly different in comparison to the ASC, Sham and CTRL groups (*P*=0.0263, *P*=0.0006, *P*=0.0002) (Table 2).

**Table 2:** The effects of ASC and ASC-CM transplantation after 4 weeks on the ovarian dimensions in the PCOS rats

Dimensions of the ovary	Groups			
	**Sham**	**CTRL**	**ASC**	**ASC-CM**
**Ovary length (mm)**	**6.927 ± 0.123**	**6.812 ± 0.057**	6.360 ± 0.157^*#^	6.388 ± 0.093^*^
**Ovary width (mm)**	**4.970 ± 0.190**	**4.744 ± 0.103**	4.337 ± 0.117^**^	4.214 ± 0.413^**#^
**Ovary weight (g)**	**0.091 ± 0.001**	**0.090 ± 0.000**	**0.096 ± 0.002**	0.103 ± 0.001^***###Φ^
**GSI (%)**	**0.028 ± 0.003**	**0.030 ± 0.001**	0.040 ± 0.002^**#^	0.040 ± 0.001^**#^

Data are expressed as the mean ± SEM and analyzed by one-way ANOVA followed by Turkeys’ post hoc test. *shows significant differences vs the Sham group (*P ≤ 0.05, **P ≤ 0.01, and ***P ≤ 0.001). ^#^ shows significant differences vs the CTRL group (^#^*P* ≤ 0.05, and ^###^*P* ≤ 0.001). ^Φ^ shows significant differences vs the ASC group (^Φ^*P* ≤ 0.05). GSI, Gonadosomatoindex; CTRL, Control group; ASC, Adipose stem cell group; ASC-CM, Adipose stem cell-conditioned media group

The GSI [(ovary weight/body weight) × 100] was calculated due to the ovarian mass variations pattern. As shown in Table 2, treatment of PCOS animals with ASC and ASC-CM significantly increased GSI parameter in the ASC-CM and ASC groups compared to that of the Sham (*P*=0.0040, *P*=0.0082) and CTRL groups (*P*=0.0103, *P*=0.0207).

### Serum estradiol assay

**Fig. 5:**
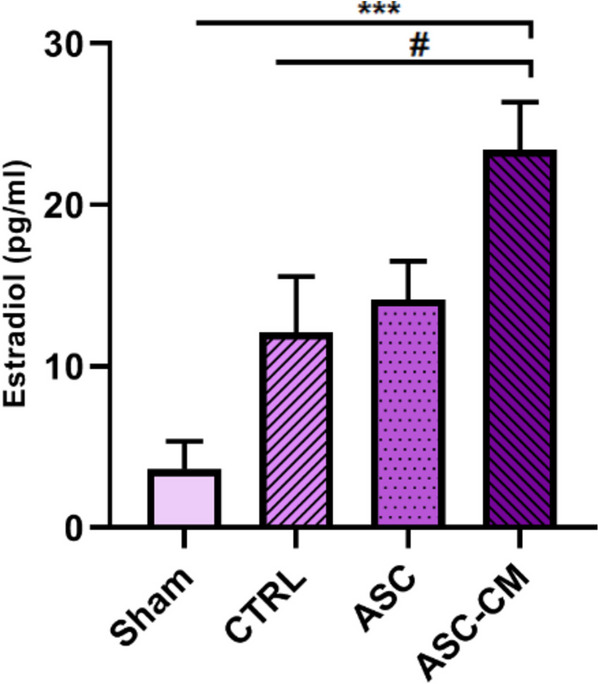
The effects of ASC and ASC-CM transplantation after 4 weeks on the serum estradiol level in different groups of PCOS rat model. *shows significant vs the Sham group (*** *P* ≤ 0.001), ^#^ shows significant differences vs the CTRL group, and (^#^*P* ≤ 0.05). Data are expressed as the mean ± SEM. Data were analyzed by one-way ANOVA followed by Tukey’s post hoc test. Abbreviations: CTRL, Control group; ASC, Adipose stem cell group; ASC-CM, Adipose stem cell-conditioned media group

As is shown in Fig. 5, a significant increase was detected in the serum estradiol level of the ASC-CM group compared to the sham (*P* = 0.0002) and CTRL (*P* = 0.0351) groups (Fig. 5).

### The Effects of ASC and ASC-CM on the follicular development in the PCOS rat model

**Fig. 6:**
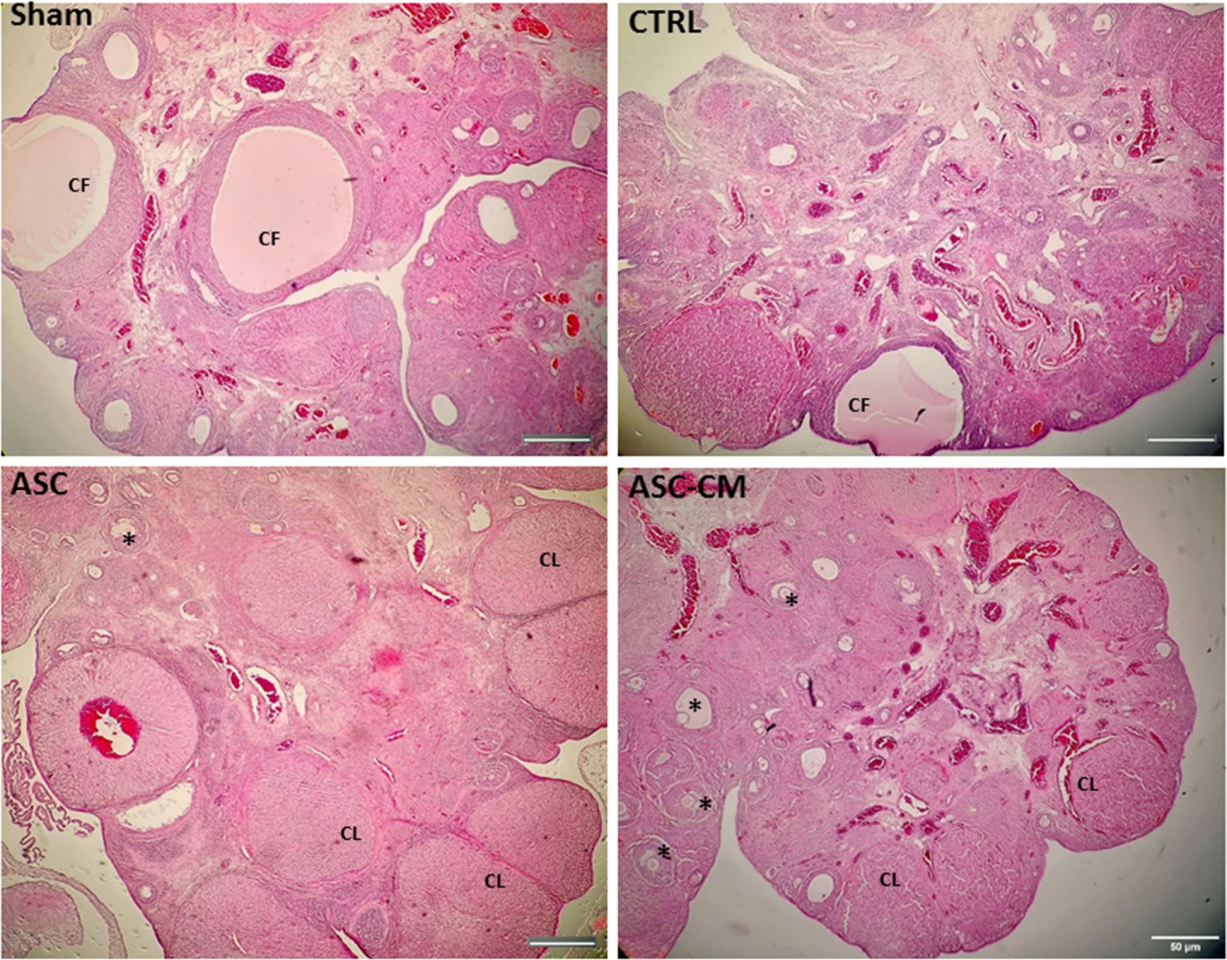
The effects of ASC and ASC-CM transplantation in the PCOS rat models after 4 weeks (hematoxylin and eosin [H&E] staining). Stars indicate the different types of follicles (scale bar = 50 µm). Abbreviations: CTRL, Control group; ASC, Adipose stem cell group; ASC-CM, Adipose stem cell-conditioned media group; CL, corpus luteum; CF, cystic follicle

As shown in Table 3, no significant changes in the mean number of primordial, secondary and pre-ovulatory follicles were observed among groups, while the mean number of primary and pre-antral follicles in the ASC-CM group was significantly decreased in comparison to the Sham (*P*<0.0001), and CTRL (*P*=0.0002) groups. Moreover, the number of primary follicles in the ASC-CM group was significantly (*P*=0.0059) less than that in the ASC group. Furthermore, the mean number of pre-antral follicles in the ASC and ASC-CM groups was less than the Sham (*P*=0.0011 and *P*<0.0001, respectively), and CTRL (*P*=0.0003 and *P*<0.0001, respectively) groups. In contrast, the mean number of antral follicles in the ASC-CM group was increased compared with the Sham and CTRL groups (*P*=0.0121, *P*=0.0499). An important finding was a significant reduction of the number of atretic follicles in the ASC and ASC-CM rats compared to the Sham (*P*=0.0031, *P*=0.0015) and CTRL (*P*=0.0008, *P*=0.0004) groups. The number of corpus luteum in the ASC-CM group was significantly higher compared to the Sham (*P*=0.0033), and CTRL (*P*<0.0001) groups. In addition, the number of corpus luteum in the ASC group was significantly increased compared to the CTRL (*P*=0.0173) group. Interestingly, ASC-CM treatment significantly decreased the number of ovarian cysts in comparison with the Sham (*P*<0.0001), CTRL (*P*=0.0006) and also ASC (*P*=0.0041) groups (Table 3) (Fig 6).

**Table 3:** The effects of ASC and ASC-CM transplantation on follicular development after 4 weeks in the PCOS rat model

Variables (number)	Groups			
	**Sham**	**CTRL**	**ASC**	**ASC-CM**
**Primordial F**	**254.0 ± 8.560**	**257.2 ± 6.590**	**250.8 ± 5.338**	**247.5 ± 5.847**
**Primary F**	**143.3 ± 4.551**	**135.0 ± 5.939**	**124.3 ± 4.201**	98.3 ± 4.551^****###ΦΦ^
**Secondary F**	**35.5 ± 1.176**	**34.0 ± 1.183**	**31.0 ± 1.317**	**31.7 ± 1.054**
**Pre-antral F**	**16.2 ± 1.014**	**16.8 ± 0.654**	10.8 ± 0.872^**###^	8.2 ± 0.749^****####^
**Antral F**	**12.8 ± 1.376**	**15.0 ± 2.463**	**20.3 ± 1.282**	24.2 ± 3.439^*#^
**Pre-ovulatory F**	**10.5 ± 0.428**	**10.2 ± 0.749**	**11.3 ± 0.615**	**11.5 ± 0.563**
**Atretic F**	**37.7 ± 4.287**	**40.5 ± 4.023**	19.0 ± 2.251^**###^	17.5 ± 1.607^**###^
**Corpus luteum**	**8.8 ± 0.924**	**6.7 ± 1.133**	**10.7 ± 0.375**^**#**^	13.7 ± 0.815^**####^
**Cysts**	**16.2 ± 1.400**	**13.2 ± 1.116**	**12.3 ± 1.085**	6.2 ± 0.703^****###ΦΦ^

Data are expressed as the mean ± SEM. *shows significant differences vs Sham (*P ≤ 0.05, **P ≤ 0.01, ***P ≤ 0.001, and ****P ≤ 0.000). ^#^shows significant differences vs CTRL group (^#^*P* ≤ 0.05, ^##^*P* ≤ 0.01, ^###^*P* ≤ 0.001, and ^####^*P* ≤ 0.000). ^Φ^ shows significant differences vs ASC group (^ΦΦ^ *P* ≤ 0.01). Data were analyzed by one-way ANOVA followed by Tukey’s post hoc test. Abbreviations: CTRL, Control group; ASC, Adipose stem cell group; ASC-CM, Adipose stem cell-conditioned media group

### The effects of ASC and ASC-CM on the estrus cycle of PCOS rat model

**Fig. 7:**
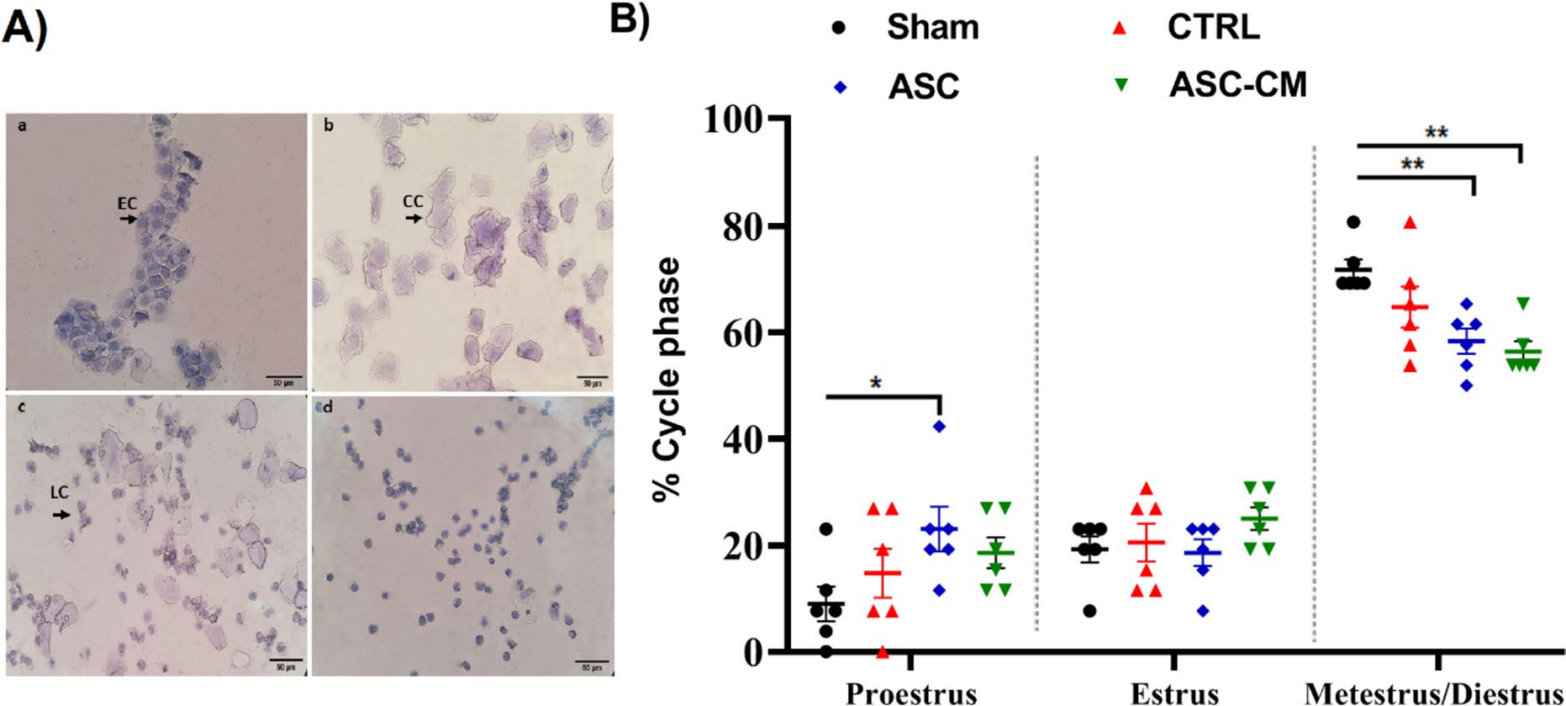
The effects of ASC and ASC-CM on the estrus cyclicity, after 4 weeks of treatment. **A** Stained slides of vaginal smears in the normal rats. **a** In the proestrus phase, the smears mainly contained epithelial cells (EC). **b** In the estrus phase, the smears contained cornified cells (CC). (**c**) In the metestrus phase, the smears contained CC, EC and leucocyte cells (LC). **d** In the diestrus phase, the smears mainly contained LC. (hematoxylin staining; magnification, × 40) (**B**) The scatterplots represent the proportion of time spent in each estrus cycle in the four groups of animals. The Y axis represents the percentile range from 0–100. * shows significant Differences vs Sham group (*P ≤ 0.05, ** *P* ≤ 0.01, and *** *P* ≤ 0.001*).* Data are expressed as mean ± SEM. Data were analyzed by one-way ANOVA followed by Tukey’s post hoc test. Abbreviations: CTRL, Control group; ASC, Adipose stem cell group; ASC-CM, Adipose stem cell-conditioned media group

The estrus cycle of *Wistar* rats is about 4 to 5 days. An example of the images of the rats’ estrus cycle is given in Fig. 7. The estrus cycle disorders were appeared after letrozole administration for 21 days as almost all the PCOS rats were in metestrus and diestrus phases. Following 4 weeks of treatment, the data showed that compared with the ASC-CM and ASC groups, the Sham group displayed disrupted estrus cycles with longer time spent in the metestrus and diestrus phases (*P* = 0.0015, *P* = 0.0048). Moreover, the proportion of proestrus stage in the ASC group significantly (*P* = 0.0419) increased compared to the Sham group. Overall, after treatment with ASC and ASC-CM, there was an increase in the proportion of proestrus and estrus stages and a decrease in the proportion of diestrus /metestrus stages in the ASC and ASC-CM groups compared to the Sham group (Fig. 7).

### The Effects of ASC and ASC-CM on IPGTT in the PCOS rat model

**Fig. 8:**
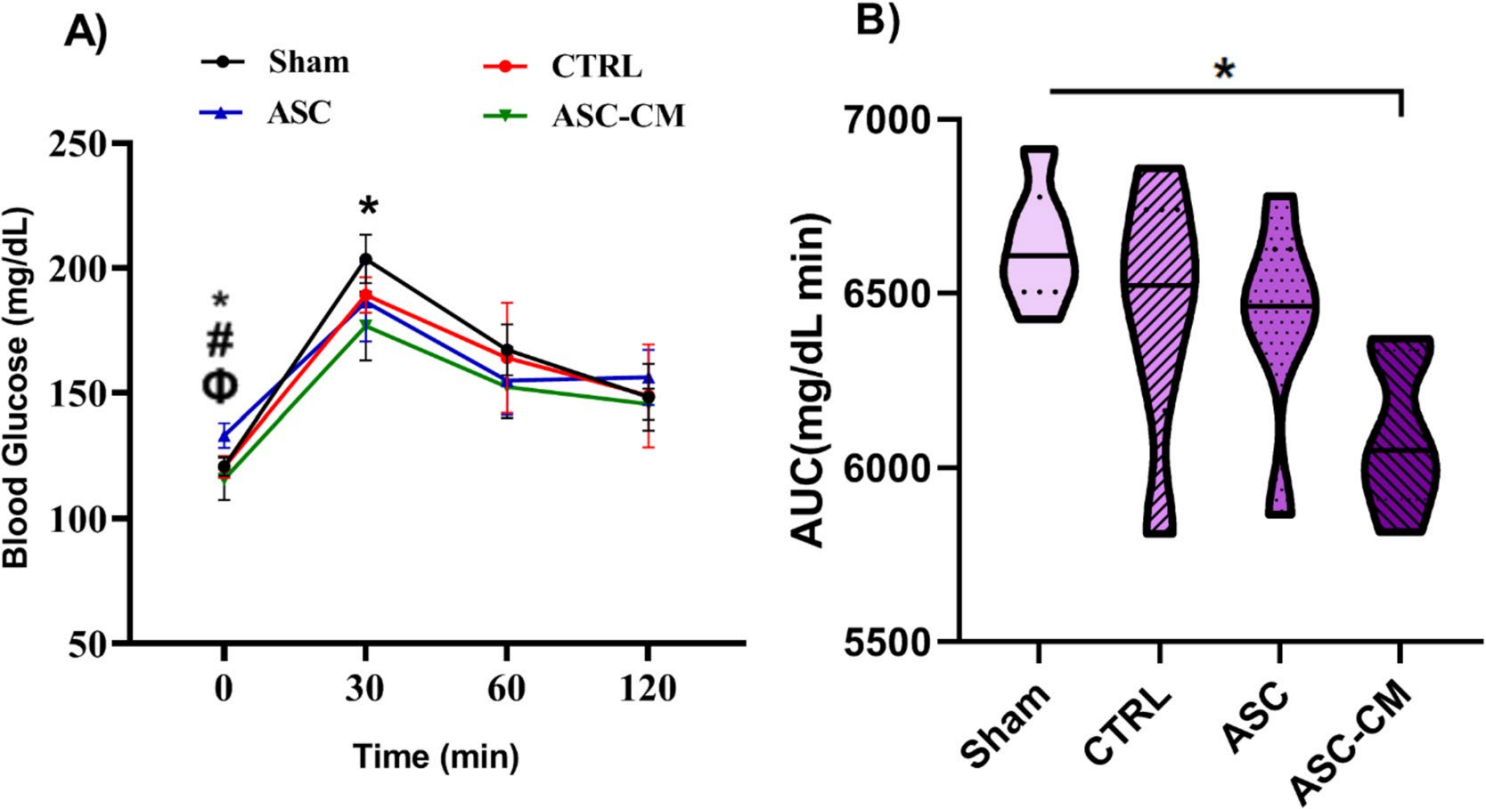
The effects of ASC and ASC-CM 4 weeks after transplantation on intraperitoneal glucose tolerance test (IPGTT) (**A**) glucose levels after 14 h fasting (time 0) and glucose levels at different times following 2 mg/dl intraperitoneal glucose injection (**B**), the area under IPGTT curves (AUC)* shows *P* ≤ 0.01, ASC vs Sham; ^#^ shows *P* ≤ 0.01, ASC vs CTRL; ^Φ^ shows *P* ≤ 0.05, ASC vs ASC-CM; at 30 min after glucose loading: * shows *P* ≤ 0.05, ASC-C.M vs Sham. **B**: *shows significant vs the Sham group (*P ≤ 0.05); Data are expressed as mean ± SEM. Repeated-measure ANOVA followed by Tukey’s post hoc test was used to analyze IPGTT data. one-way ANOVA followed by Tukey’s post hoc test was used for AUC data analysis Abbreviations: CTRL, Control group; ASC, Adipose stem cell group; ASC-CM, Adipose stem cell-conditioned media group

Four weeks after treatment, IPGTT was done at different times (0, 30, 60 and 120 min). Also, the values of the areas under the blood glucose curves during the 2-h tolerance test were calculated as AUC (Fig. 8.A, B).

Glucose concentration varied significantly with time [F (2.667, 53.34) = 112.8, *P* < 0.0001] and group [F (3, 20) = 5.877, *P* = 0.0048]. Before the administration of glucose, a significant difference was found in the glucose level between the studied groups (Fig. 8.A). Fasting blood sugar in the ASC group was significantly higher than the Sham (*P* = 0.0037), CTRL (*P* = 0.0044) and ASC-CM (*P* = 0.0110) groups. Interestingly, thirty minutes after glucose loading, the ASC-CM group had a significantly (*P* = 0.0154) lower glucose level than the Sham group. Furthermore, the ASC-CM group had a significantly (*P* = 0.0138) lower AUC level compared to that of the Sham group (Fig. 8.B).

### The effects of ASC and ASC-CM on the expression level of DNA methylation and histone deacetylation regulators in the ovary of PCOS rat model

**Fig. 9:**
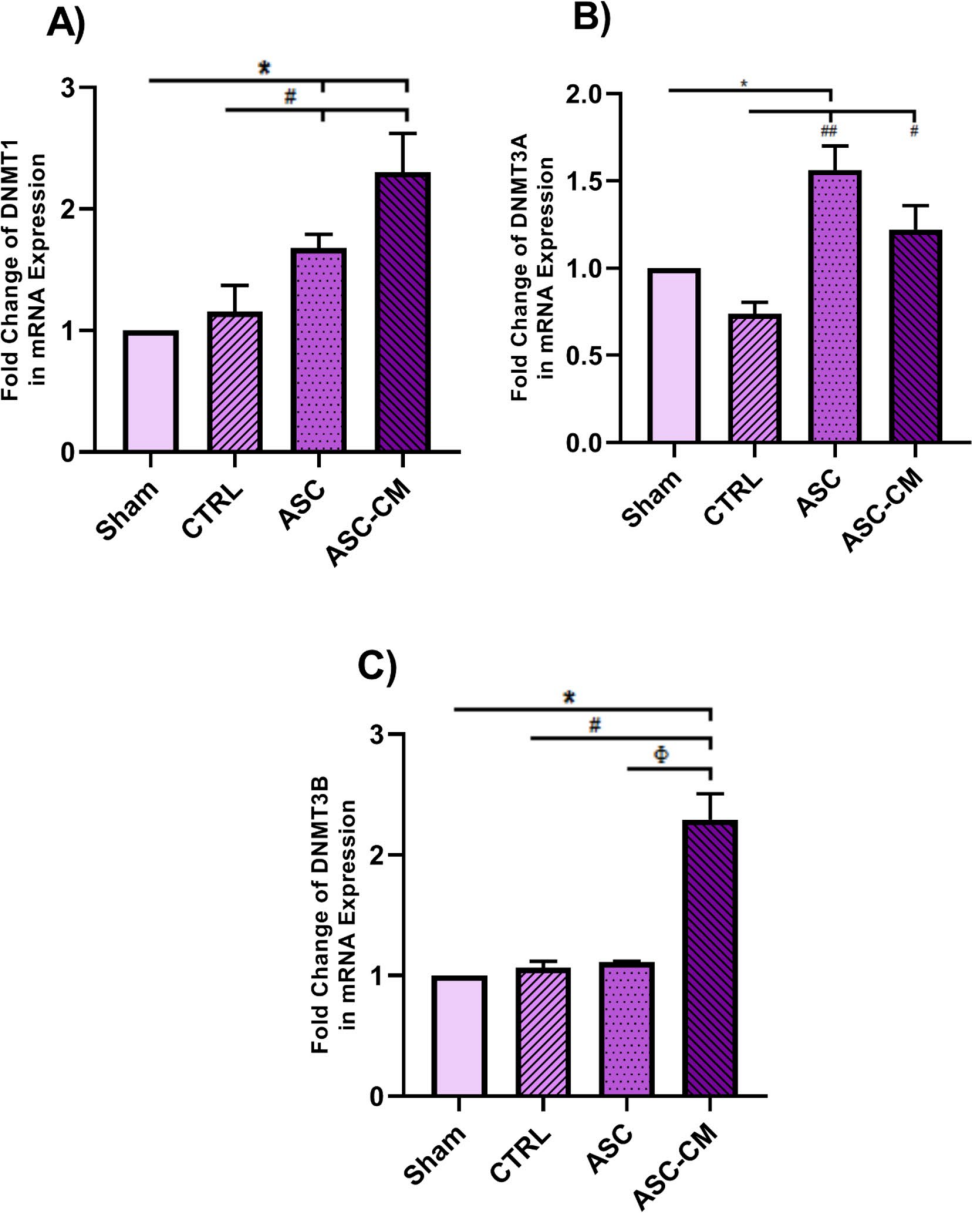
The effects of ASC and ASC-CM transplantation on the expression levels of *DNMT1* (**A**), *DNMT3A* (**B**) and *DNMT3B* (**C**) four weeks after treatments. The mRNA level of each sample was normalized against β-actin. Values represent the means ± SEM from each group. Data were analyzed by Kruskal–Wallis test and one-way ANOVA followed by Tukey’s post hoc test. *shows significant differences vs the Sham (*P ≤ 0.05). ^#^shows significant differences vs the CTRL group (^#^*P* ≤ 0.05, and ^##^*P* ≤ 0.01). ^Φ^ shows significant differences vs ASC group (^Φ^ *P* ≤ 0.05). Abbreviations: CTRL, Control group; ASC, Adipose stem cell group; ASC-CM, Adipose stem cell-conditioned media group

The fold change of the epigenetic modification enzymes *DNMT1, DNMT3A, DNMT3B, HDAC1,* and *HDAC2* in the right ovary was determined using qRT-PCR. As shown in Fig. 9. A, a significant up-regulation in *DNMT1* expression was observed in the ASC and ASC-CM groups compared sham (*P* = 0.037) and CTRL(*P* = 0.05) groups.

Moreover, the *DNMT3A* level was significantly higher in the ASC group compared to the Sham (*P* = 0.022) and CTRL (*P* = 0.002) groups. It was also significantly (*P* = 0.05) higher in the ASC-CM group than the CTRL group (Fig. 9.B).

In addition, there was a significant increase in the expression level of *DNMT3B* in the ASC-CM group in comparison to the sham (*P* = 0.037), ASC and CTRL groups (*P* = 0.05) (Fig. 9.C).

**Fig. 10:**
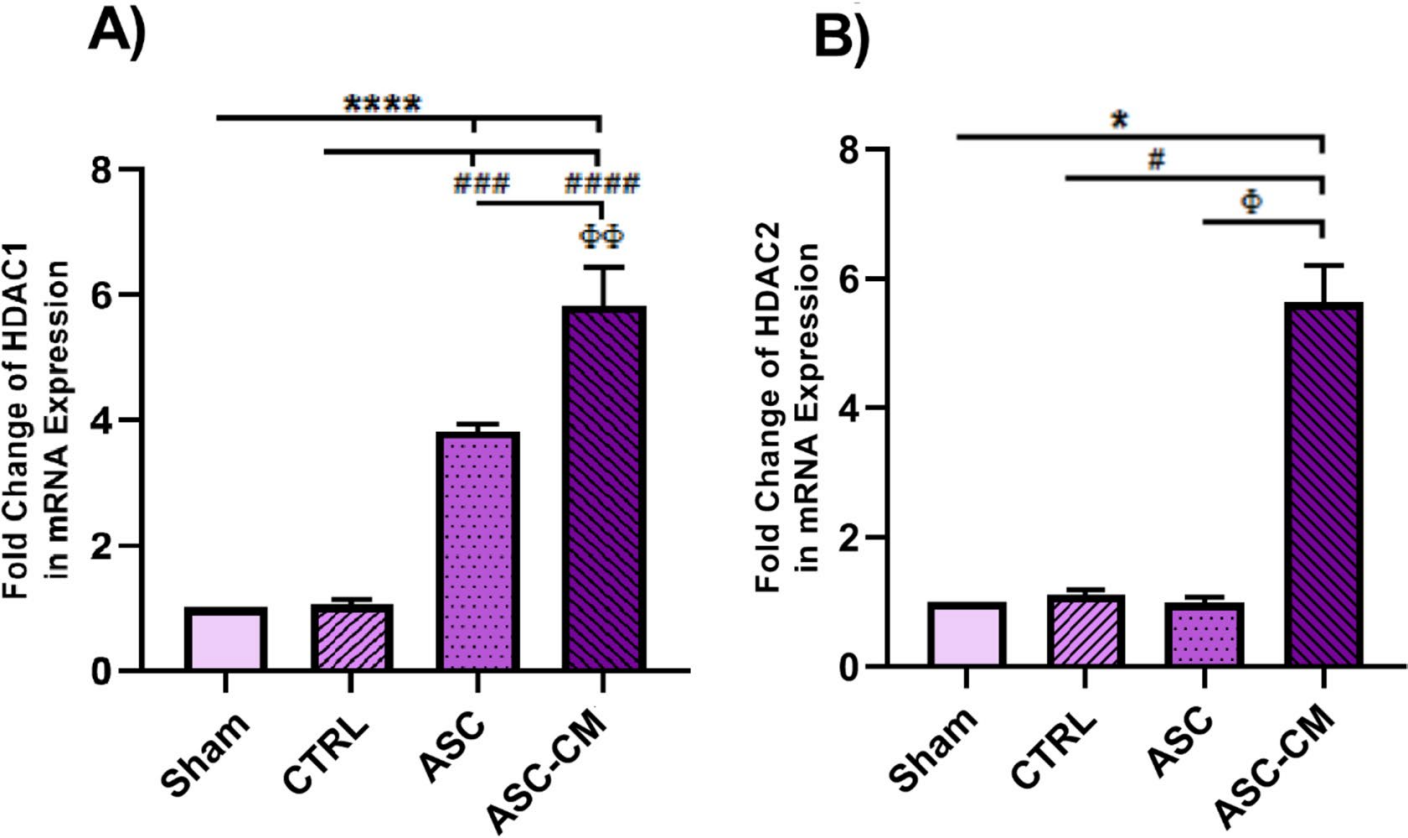
The effects of ASC and ASC-CM transplantation on the expression of *HDAC1* (**A**) and *HDAC2* (**B**) after 4 weeks. The mRNA level of each sample was normalized against β-actin. Values represent the means ± SEM from each group. Data were analyzed by Kruskal–Wallis test and one-way ANOVA followed by Tukey’s post hoc test*.* *shows significant differences vs Sham (*P ≤ 0.05, and ****P ≤ 0.000). ^#^shows significant differences vs CTRL group (^#^ *P* ≤ 0.05, ^###^ *P* ≤ 0.001, and ^####^*P* ≤ 0.000). ^Φ^ shows significant differences vs ASC group (^Φ^ *P* ≤ 0.05, and ^ΦΦ^ *P* ≤ 0.01). Abbreviations: CTRL, Control group; ASC, Adipose stem cell group; ASC-CM, Adipose stem cell-conditioned media group

As shown in Fig. 10.A, transplantation of ASC-CM significantly increased the expression of *HDAC1* gene in the ASC-CM group compared to that of the Sham (*P* = 0.000), CTRL (*P* = 0.000) and ASC (*P* = 0.006) groups. Moreover, the expression of *HDAC1* gene in the ASC group was significantly increased compared to the Sham (*P* = 0.000) and CTRL (*P* = 0.001) groups.

Transplantation of ASC-CM to the ovary, resulted in a significant increase in the expression of *HDAC2* compared to that of the sham (*P* = 0.037), ASC and CTRL groups (*P* = 0.05) (Fig. 10.B). Overall, our data indicated that the highest expressions of *HDAC1* and *HDAC2* were found in the ASC-CM group rather than others (Fig. 10.A, B).

### The effects of ASC and ASC-CM on the expression level of estrogen receptors in the ovary of PCOS rat model

**Fig. 11:**
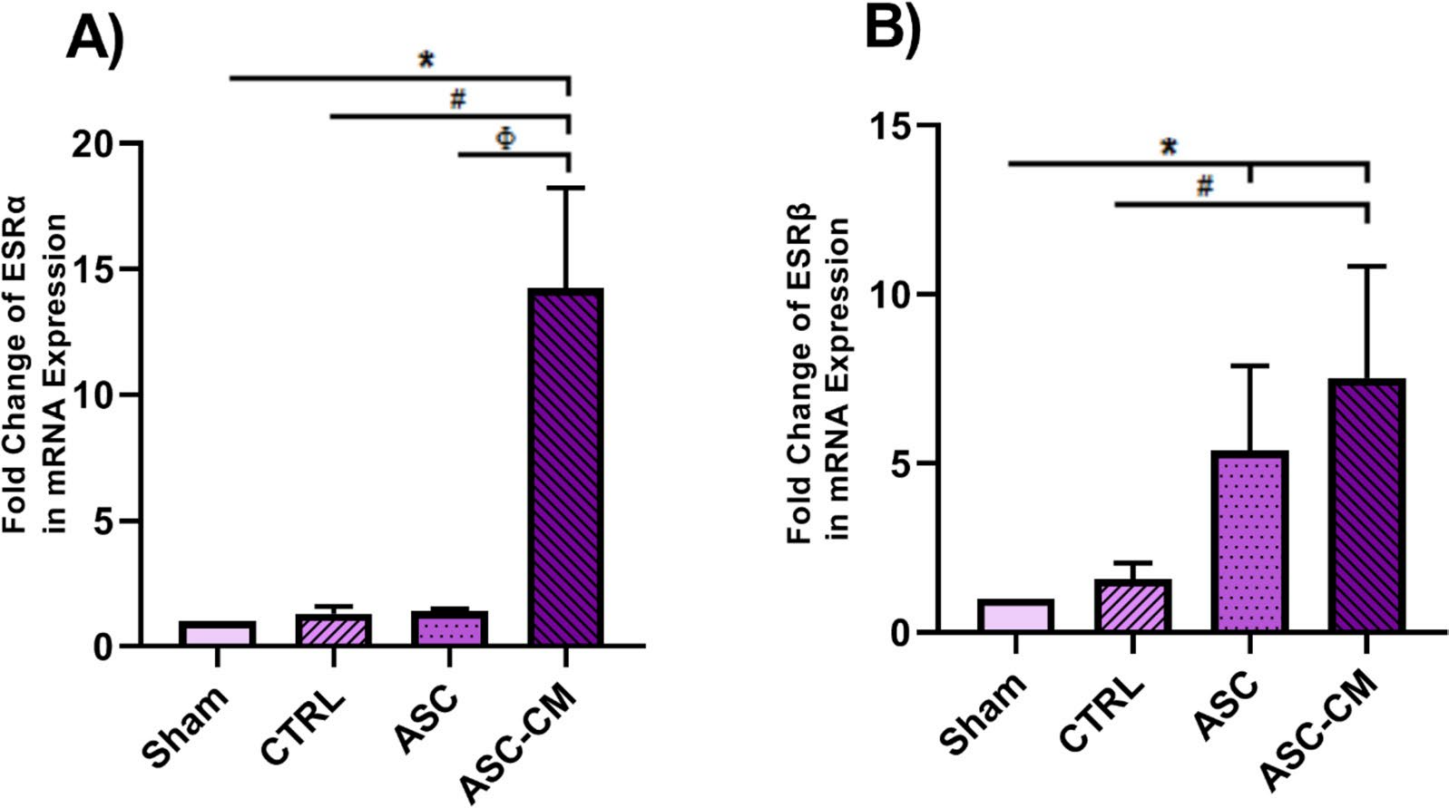
The effects of ASC and ASC-CM transplantation on the expression of *ESRα* (**A**) and *ESRβ* (**B**) after 4 weeks. The mRNA level of each sample was normalized against *β-actin*. Values represent the means ± SEM from each group. Data were analyzed by Kruskal–Wallis test*.* *shows significant differences vs Sham (*P ≤ 0.05). ^#^shows significant differences vs CTRL group (#*P* ≤ 0.05). ^Φ^ shows significant differences vs ASC group (^Φ^ *P* ≤ 0.05). Abbreviations: CTRL, Control group; ASC, Adipose stem cell group; ASC-CM, Adipose stem cell-conditioned media group, ESRα, Estrogen Receptor α; ESRβ, Estrogen Receptor β

The impact of ASC and ASC-CM on the expression levels of key genes implicated the esteroidal actions; *ESRα* and *ESRβ* were examined in the right ovary. As shown in Fig. 11, ASC-CM transplantation significantly increased the expression of *ESRα* in the ASC-CM group compared to that of the sham (*P* = 0.037), ASC and CTRL (*P* = 0.05) (Fig. 11.A). In parallel with this result, a significant increase in the mRNA expression of *ESRβ* was also evident in the ASC-CM group compared to the Sham (*P* = 0.037) and CTRL (*P* = 0.05) groups (Fig. 11.B). In addition, a significant (*P* = 0.037) overexpression of *ESRβ* was detected in the ASC group compared to the Sham group (Fig. 11.B).

### The Effects of ASC and ASC-CM on the 5mCand 5hmC protein levels in the left ovary of PCOS rat model

To evaluate the levels of 5mC and 5hmC in the ovary of the animals, the immunohistochemistry method was used and the images were quantitated using the ImageJ software (TotalLab Quant, UK).

**Fig. 12:**
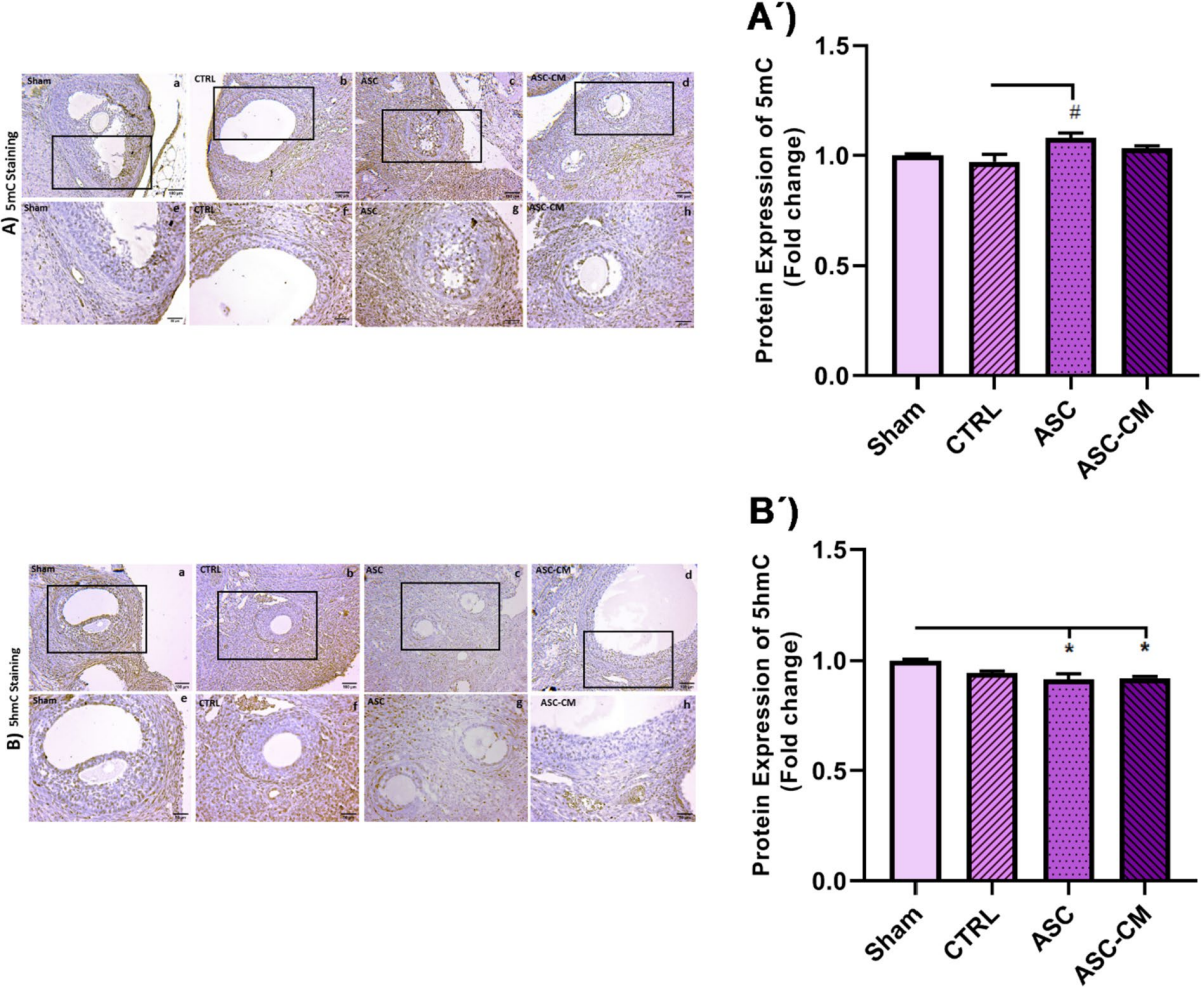
The effects of ASC and ASC-CM transplantation on 5mC (A,A**´**) and 5hmC (B,B´) nuclear protein levels 4 weeks after treatment in the left ovary of PCOS rat model. Values represent the means ± SEM from each group. One-way ANOVA followed by Tukey’s post hoc test was used to analyze data. Positive cells (brown staining) were detected in both the parenchymal and luteal structures of the ovaries. *shows significant differences vs Sham (*P ≤ 0.05). ^#^shows significant differences vs CTRL group (.^#^*P* ≤ 0.05). Abbreviations: CTRL, Control group; ASC, Adipose stem cell group; ASC-CM, Adipose stem cell-conditioned media group; 5mC, 5methyl-cytosine; 5hmC, 5-Hydroxymethylcytosine; SEM, standard error of the mean. Magnification and Scale bars =  × 100; 100 µm (**a**–**d**) and × 200; 50 µm (**e–h**)

As shown in Fig. 12.A and A´, the ovary of rats in the ASC group displayed a significant increase in the protein expression level of 5mC in comparison to the CTRL group (*P* = 0.0263). Notably, the protein level of 5hmC was significantly decreased in the ASC (*P* = 0.0163) and ASC-CM (*P* = 0.0258) groups versus the Sham group (Fig. 12.B, B´).

### The Effects of ASC and ASC-CM on the protein levels of estrogen receptors in the ovary of PCOS rat model

**Fig. 13:**
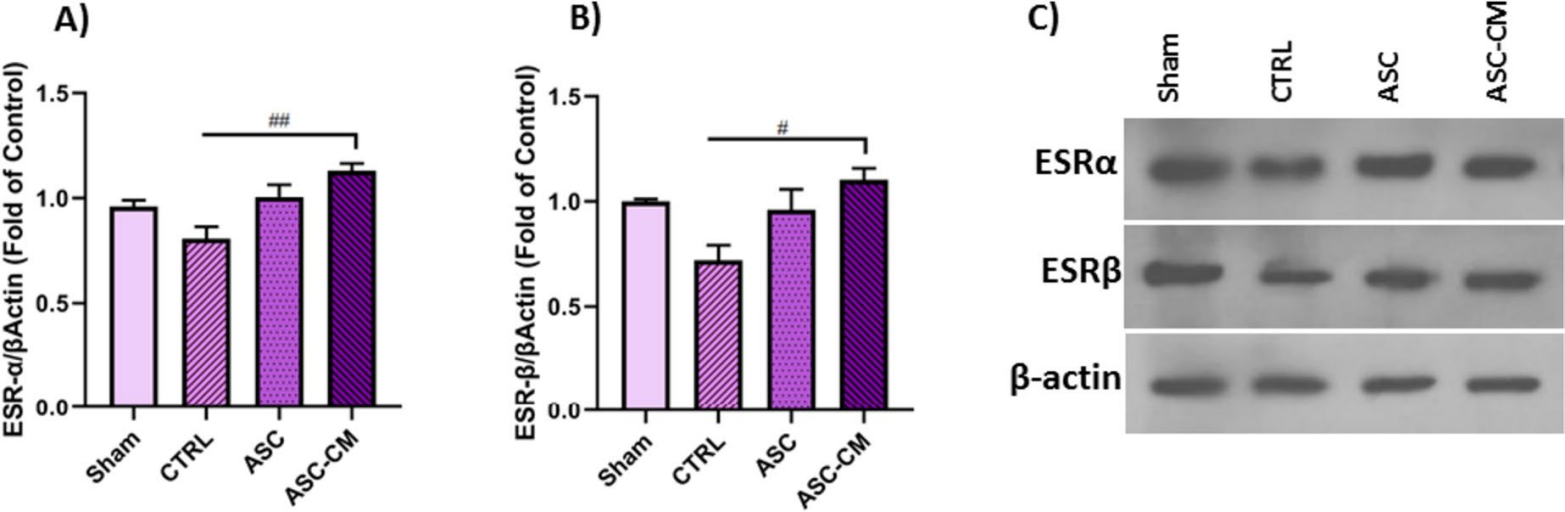
The effects of ASC and ASC-CM transplantation on the ESRα (**A**) and ESRβ (**B**) protein level after 4 weeks in the right ovary of PCOS rat model. **C** ESRα and ESRβ bands in the ovary of PCOS rat model. Values represent the means ± SEM of each group. One-way ANOVA followed by Tukey’s post hoc test was used to analyze data. β-Actin was used as an internal loading control. ^#^shows significant Differences between groups (^#^*P* ≤ 0.05, ^##^ *P* ≤ 0.01). Abbreviations: CTRL, Control group; ASC, Adipose stem cell group; ASC-CM, Adipose stem cell-conditioned media group; ESRα, Estrogen Receptor α; ESRβ, Estrogen Receptor β

To determine the effects of ASC and ASC-CM transplantation after 4-weeks on the ESRα and ESRβ protein levels (Fig. 13.A-C) western blot analysis was done. The results revealed a significant increase in the protein level of the ESRα and ESRβ in the ASC-CM group compared to the CTRL (*P* = 0.0056, *P* = 0.0160 respectively) group (Fig. 13.A and C). (Fig. 13. B and C).

## Discussion

We showed that ASC-CM, compared with ASC, has more positive effects on folliculogenesis and estradiol function in a PCOS rat model via the involvement of key epigenetic regulators.

Previous studies showed that DNA hypomethylation occurs in various candidate genes implicated in PCOS pathogenesis [46]. Hence, we observed decreased expression of DNA methylation regulators including DNMT1, DNMT3A, and DNMT3B in the ovarian tissue of untreated PCOS rats (sham and CTRL animals). Additionally, the PCOS ovaries showed a 5mC decrease in the genome methylation index and a 5hmC rise in the genome demethylation index. According to a prior research, PCOS women's GCs had 25% lower 5mC levels than the control group [47]. Therefore, a decline in the total content of DNA methylation and DNMT1 expression was observed in the oocytes of PCOS mouse model [48]. Sagvekar et al. [49] revealed that the downregulation of DNMT3A may contribute to DNA methylation changes in the GCs of PCOS women. Moreover, in line with DNA hypomethylation, we observed aberrant histone acetylation patterns in the untreated PCOS animals that are associated with epigenetic signature modification [50]. The PCOS mouse oocyte has been seen to have a reduction in HDAC1 expression, as well as a decrease in DNA and histone methylation. This phenomenon has been linked to elevated plasma androgen levels [48]. Thus, the PCOS animal model with prenatal testosterone exposure showed a significant decrease in HDAC3 gene expression in TCs of the ovary [15]. The changes in the PCOS ovaries might affect the expression level of important genes involved in PCOS pathogenesis. For example, reduced histone deacetylation [51] and DNA methylation [46] in PCOS patients can affect gene expression involved in androgen production, resulting in hyperandrogenism. DNA hypomethylation and reduced histone deacetylation can increase the expression of LH receptors in GCs and TCs [46, 52], leading to higher P450C17a activity. P450C17a is a key enzyme in androgen synthesis, and an increase in its activity or expression promotes excessive androgen synthesis in the ovary [53]. Furthermore, since DNMT1 and HDAC1 are two critical genes for proper epigenetic modification and development, changes to these genes may impact the results of maturation, fertilization, and embryonic development in PCOS mice [54], ultimately leading to infertility. The expression of DNMTs and HDACs increased in the ovaries of treated PCOS rats, especially in those animals that received ASC-CM. Moreover, higher expression of 5mC and lower expression of 5hmC were observed in treated ovaries. It appears that microRNAs in exosome-derived MSCs may reprogram cells to repair damage and mediate protection by regulating the epigenetic process [55], so examining the exosomes in the CM may provide additional information. Furthermore, further research is necessary to validate the possibility that ASC-CM is linked to epigenetic modification-induced gene silencing in the promoter region of genes relevant to PCOS [56, 57]. Totally, the upregulation of DNMTs, HDACs, along with higher levels of 5mC in the ASC and ASC-CM groups may be related to hypermethylation of some important genes that play a pivotal role in the pathogenesis of PCOS which can control the side effects of PCOS [46].

Aberrant epigenetic status may alter the expression of genes involved in steroid synthesis, thereby affecting estrogen production, and ovarian function [12, 58, 59]. Estrogen may alter folliculogenesis by increasing the sensitivity of GCs to FSH and LH, promoting their proliferation in primary and pre-antral follicles, and modifying the production of progestins and cAMP [4]. This hormone mediates its effects via three types of receptors. The genomic pathway via ESRα and ESRβ, and the non-genomic pathway via G-protein-coupled estrogen receptor (GPER) [60]. Letrozole-induced PCOS rats were reported to downregulate ESRα and ERβ mRNA expression, which could be involved in abnormal folliculogenesis in PCOS patients [4, 61, 62]. In alignment with the aforementioned results, our study revealed a significant decrease in the expression of estrogen receptors and serum estradiol levels in the untreated mice with PCOS. The inhibition of ESRα and ESRβ in the PCOS mouse model has resulted in ovarian cysts, hemorrhagic follicles, elevated androgen, and LH levels, as well as folliculogenesis and ovulatory failure [60]. However, after treatment of animals with ASC-CM, in our study, the expression of ESRα and ESRβ genes and proteins increased, along with the serum estradiol level. These changes may affect the hypothalamic-pituitary–gonadal (HPG) axis resulting in higher secretion of serum estradiol and improvement of ovarian function, leading to subsequent fertility in PCOS patients [6]. Thus, it was reported that ASC may suppress androgen production and stimulate steroidogenic gene expression in a PCOS rat model.[28].

Our results showed impaired folliculogenesis in the untreated PCOS rats, which was evident in a notable increase in the number of primary and pre-antral follicles, atretic, and cystic follicles as well as a decrease in the number of antral follicles and corpus luteum. These findings are consistent with prior reports in PCOS animal models [63, 64]. These events were shown to be associated with an altered hormonal profile, such as ovarian androgen overproduction in PCOS animals leading to the stimulation of small follicles development and reduction of FSH secretion [65, 66]. Tyndall et al. demonstrated that in immature rats, testosterone plays a pivotal role in GCs atresia, which leads to a reduction in the number of antral follicles [59]. However, ASC and ASC-CM transplantation improved folliculogenesis by maturation of greater numbers of small follicles without growing arrest, as well as improving the occurrence of ovulation by increasing the number of corpus luteum. The corpus luteum is the main source of progesterone secretion which is responsible for the regulation of reproductive cycles, as well as the preparation of uterus for conception [67]. As a result, an increase in corpus luteum numbers indicates that PCOS rats' ability to conceive may have improved. To help PCOS patients' ovaries heal, MSCs [63] and their exosome [68] may control follicular maturation, decrease GC apoptosis and promote their proliferation, as well as boost the production of growth factors [68, 69]. MSC-CM has a notable capacity for suppression of androgen release and the regulation of steroidogenesis within in vitro and in vivo PCOS models [70]. Meanwhile, using ASCs and their CM have uncovered their potential to increase embryo development and oocyte maturation [31]. Secreted growth factor in CM could regulate signaling pathways associated with androgen synthesis via suppressing androgen production and downregulation of androgen-synthesizing gene expression [31]. Therefore, CMs enhance ovarian function through the modulation of epigenetic regulators [71]. Further investigations into these epigenetic mechanisms is necessary to fully understand and use these regenerative properties for clinical applications.

It has been shown that metabolic problems in PCOS humans and animal models are associated with ovarian function [72]. In line with earlier findings in PCOS patients [73] and animal models [74], our results revealed a substantial rise in the ovarian dimensions, body weight gain, longer diestrus phase, and increased glucose levels in PCOS rats. These changes can be attributed to letrozole-induced hormonal disorders and epigenetic modification [75]. Hormonal disturbances are in line with obesity [76], higher blood glucose levels and increased ovary dimensions [67, 77], as well as irregular estrus cycle in the PCOS animal models [78]. Rajan et al. showed that the prolonged diestrus phase and increased body weight are related to increased testosterone concentration [79]. Furthermore, a significant improvement in the ovarian parameters, estrus cycle, and glucose levels was detected in the ASC-CM-treated rats, which is in agreement with the Jahan et al. reports [80]. Exosomes present in a conditioned media have the capacity to modulate sex hormone profiles [81], inflammation linked with obesity, and metabolic diseases [82]. Another possible mechanism involved in these positive effects of ASC-CM might be its ability to downregulate genes responsible for adiposity, and imbalance hormone profiling by modification of epigenetic regulators [56, 81].

In conclusion, our study determined that changes in the 5mC and 5hmC protein levels along with alterations in the epigenetic regulators such as DNMTs and HDACs, as well as estrogen receptors α and β, and the reduction of serum estradiol levels are related to the PCOS pathogenesis which can be recovered via ASC and ASC-CM administration. Furthermore, by changing the expression of estrogen receptors in rats with PCOS, ASC-CM may restore steroid function and folliculogenesis. Additionally, it offers fresh proof of how DNMTs, HDACs, and ESR α and β are regulated during this process, which PCOS sufferers should research.

### Supplementary Information

**Additional file 1.**

## Data Availability

The data used to support the finding of current study are available from the corresponding author, on reasonable request**.**
