# Supplementary material for: Administration of adipose-derived mesenchymal stem cell conditioned medium improves ovarian function in polycystic ovary syndrome rats: involvement of epigenetic modifiers system
Source: J Ovarian Res. 2023 Dec 15;16:238. doi: 10.1186/s13048-023-01317-9 (PMC10722730; doi:10.1186/s13048-023-01317-9)
Supplement: Supplementary file 1 — Additional file 1. [file 13048_2023_1317_MOESM1_ESM.zip › Comments to WB images.docx]

Comments

There are 5 lanes in the colored and grayscale original images while there are 4 lanes in the final picture. The first lane was for another treatment not included in the present manuscript. Therefore, that lane was deleted in the final picture.
